# Supplementary material for: Lattice pinning in MoO3 via coherent interface with stabilized Li+ intercalation
Source: Nat Commun. 2023 Oct 20;14:6662. doi: 10.1038/s41467-023-42335-x (PMC10589268; doi:10.1038/s41467-023-42335-x)
Supplement: Supplementary file 1 — Supplementary Information [file 41467_2023_42335_MOESM1_ESM.pdf]

Supplementary Information

Lattice pinning in MoO_3 via coherent interface with stabilized Li^+ intercalation

Shuo Sun,[1] Zhen Han,[2] Wei Liu,[1] Qiuying Xia,[1] Liang Xue,[1] Xincheng Lei,[2] Teng Zhai,[1] Dong Su,[2*] and Hui Xia [1*]*

Correspondence to Hui Xia (email: xiahui@njust.edu.cn), or Dong Su (email: dongsu@iphy.ac.cn), or Teng Zhai (email:tengzhai@njust.edu.cn).

Supplementary Figures

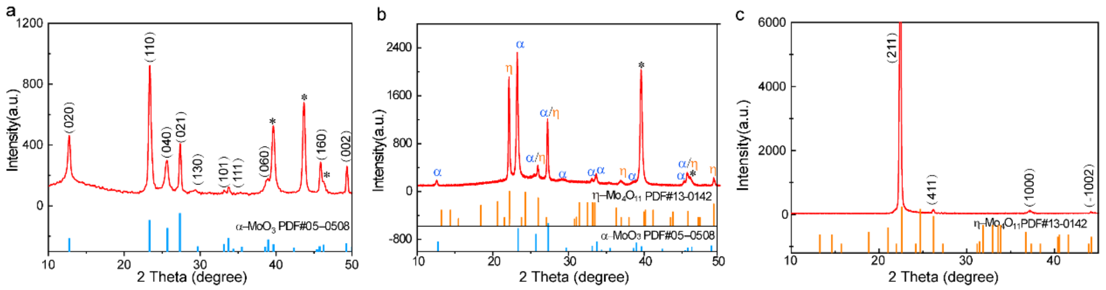

Supplementary Figure 1 XRD patterns with intensity data. (a) α-MoO_3 (α), (b) α-MoO_3/η-Mo_4O_{11} (α/η), and (c) η-Mo_4O_{11} (η) thin films. The asterisk represents the substrate.

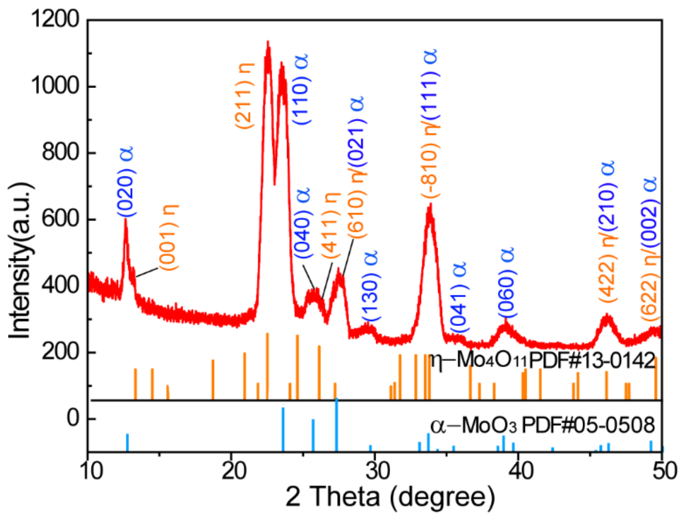

Supplementary Figure 2 The GIXRD patterns of α-MoO_3/η-Mo_4O_{11} (α/η) thin films with intensity data.

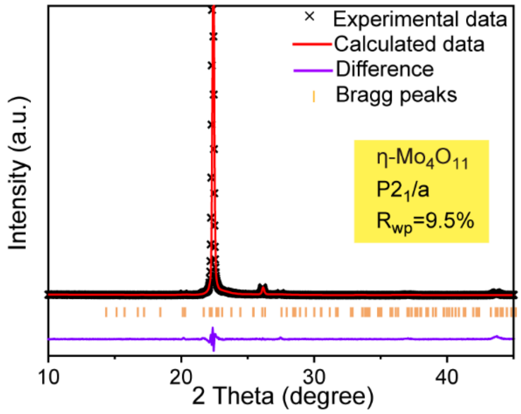

Supplementary Figure 3 Rietveld-refined XRD pattern of the η-Mo_4O_{11} thin film.

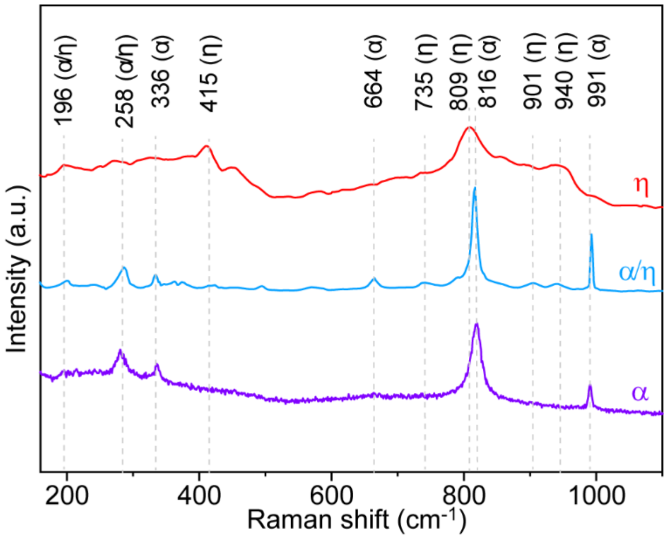

Supplementary Figure 4 Raman spectra of the η-Mo_4O_{11} (η), α-MoO_3/η-Mo_4O_{11} (α/η), and α-MoO_3 (α) thin films.

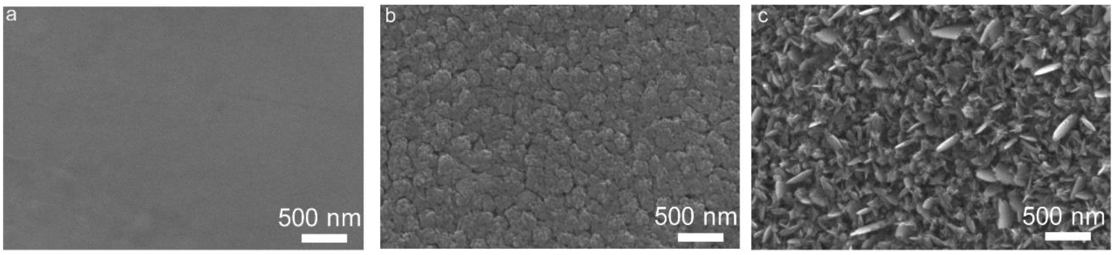

Supplementary Figure 5 Morphology of thin films. FESEM images of (a) η-Mo_4O_{11}, (b) α-MoO_3/η-Mo_4O_{11}, and (c) α-MoO_3 thin films.

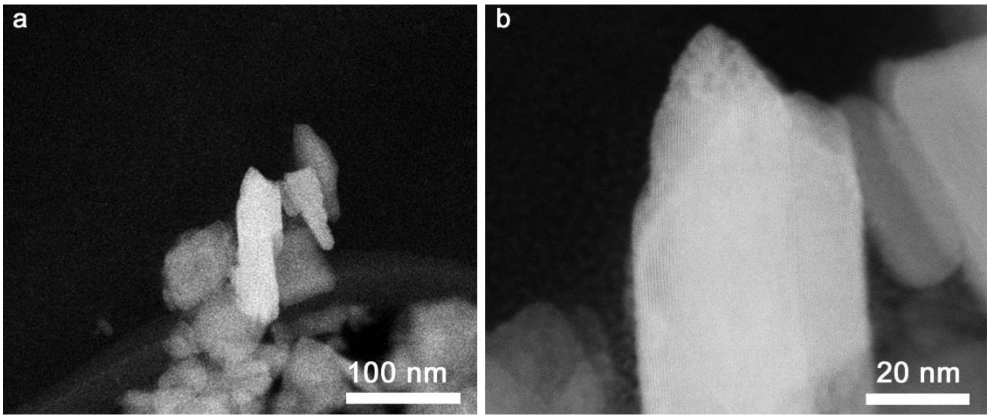

Supplementary Figure 6 Morphology of the α-MoO_3/η-Mo_4O_{11} sample. (a–b) HAADF-STEM images for the α-MoO_3/η-Mo_4O_{11} sample at different magnifications.

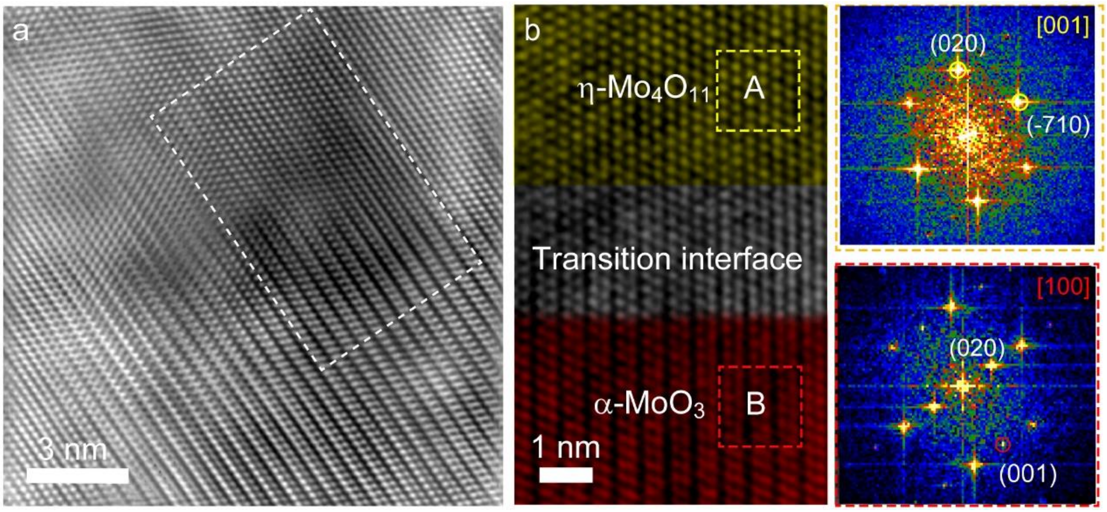

Supplementary Figure 7 The nanostructure of α-MoO_3/η-Mo_4O_{11}. (a) HRTEM image for the α-MoO_3/η-Mo_4O_{11} sample. (b) HRTEM image for interface region of the α-MoO_3/η-Mo_4O_{11} sample along the [100] zone axis of α-MoO_3. (Upper right) FFT images for monoclinic η-Mo_4O_{11} (collected from A area) of the α-MoO_3/η-Mo_4O_{11} sample along the [001] zone axis. (Lower right) FFT images for layered α-MoO_3 (collected from B area) of the α-MoO_3/η-Mo_4O_{11} sample along the [100] zone axis.

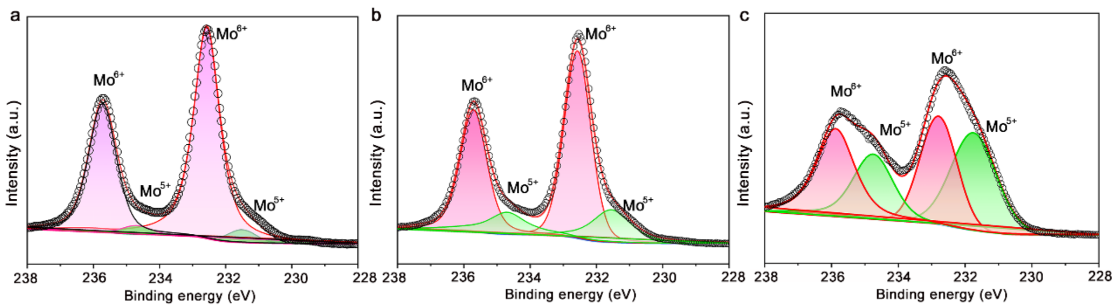

Supplementary Figure 8 Mo *3d* core-level XPS spectra. (a) α-MoO_3, (b) α-MoO_3/η-Mo_4O_{11}, and (c) η-Mo_4O_{11} thin films.

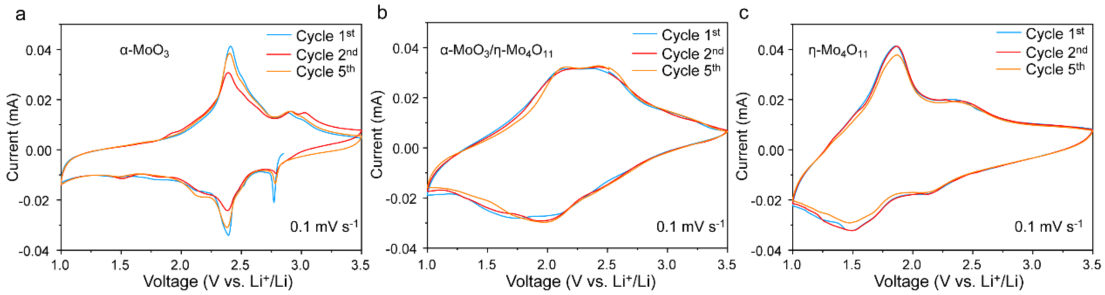

Supplementary Figure 9 Cyclic voltammograms (CV) analysis. The CV curves of (a) α-MoO_3, (b) α-MoO_3/η-Mo_4O_{11}, and (c) η-Mo_4O_{11} electrodes at the 1st, 2nd, and 5th cycles, respectively.

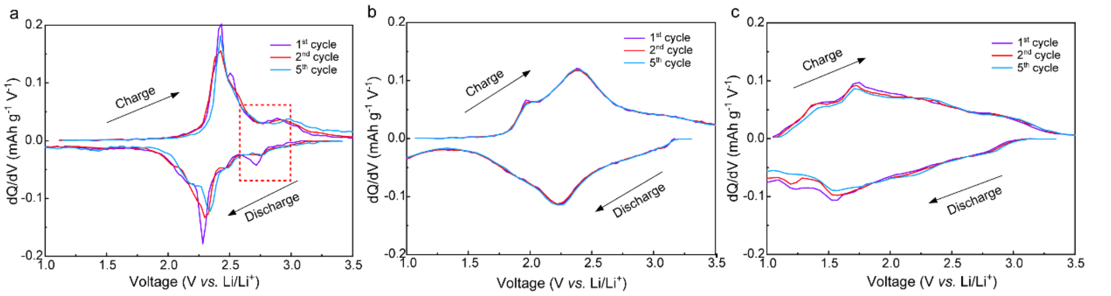

Supplementary Figure 10 Calculated dQ/dV profiles for the 1st, 2nd, and 5th cycles at the specific current of 0.1 A g^{-1}. (a) α-MoO_3, (b) α-MoO_3/η-Mo_4O_{11}, and (c) η-Mo_4O_{11} electrodes.

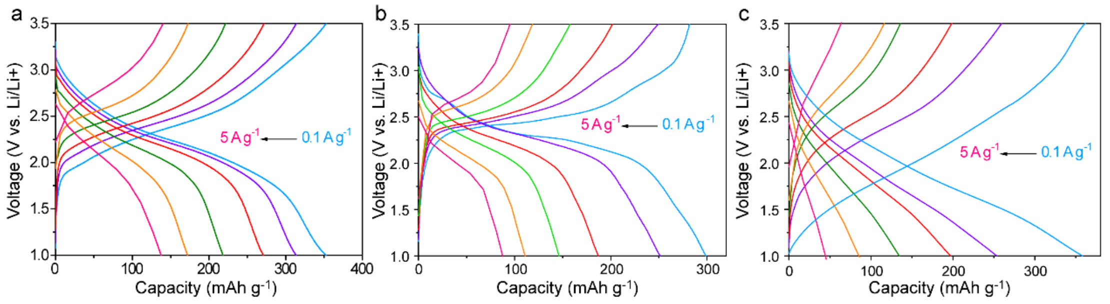

Supplementary Figure 11 Galvanostatic charge/discharge profiles at the different specific currents. (a) α-MoO_3/η-Mo_4O_{11}, (b) α-MoO_3, and (c) η-Mo_4O_{11} electrodes.

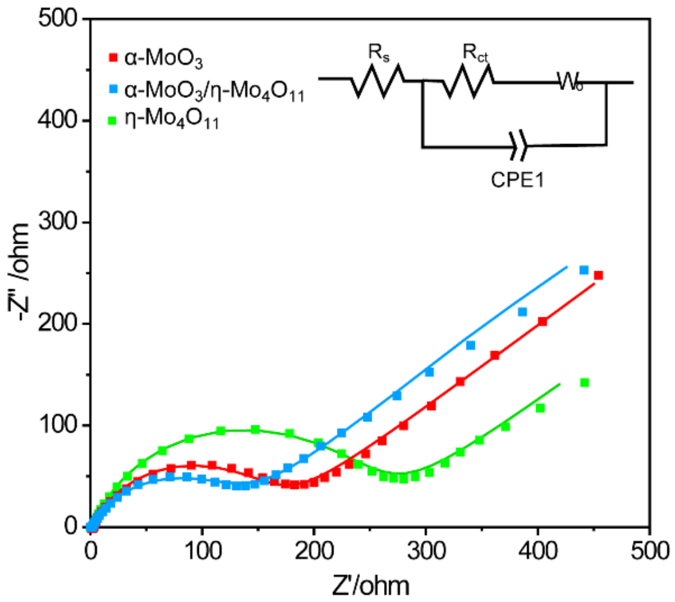

Supplementary Figure 12 Fitted EIS patterns of α-MoO_3/η-Mo_4O_{11}, α-MoO_3, and η-Mo_4O_{11} electrodes. The symbols and solid lines show the experimental and the fitted data, respectively. Inset is the equivalent circuit model used to fit the EIS data.

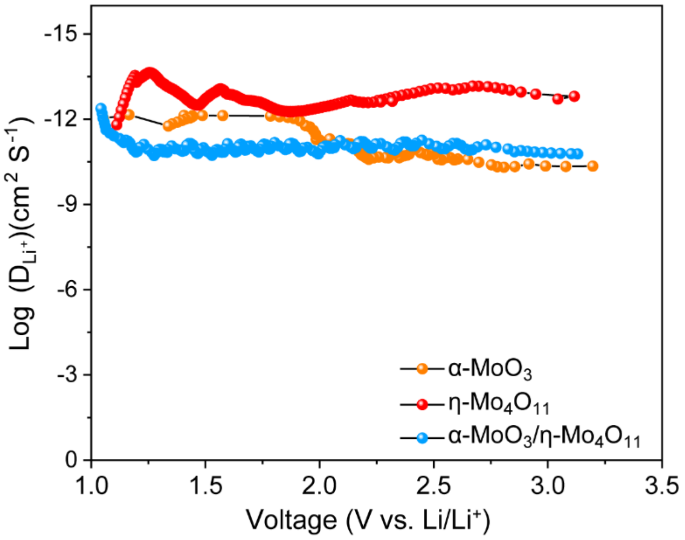

Supplementary Figure 13 Chemical diffusion coefficients of Li^+ (D_{Li^+}) of α-MoO_3/η-Mo_4O_{11}, α-MoO_3, and η-Mo_4O_{11} electrodes during the lithiation process.

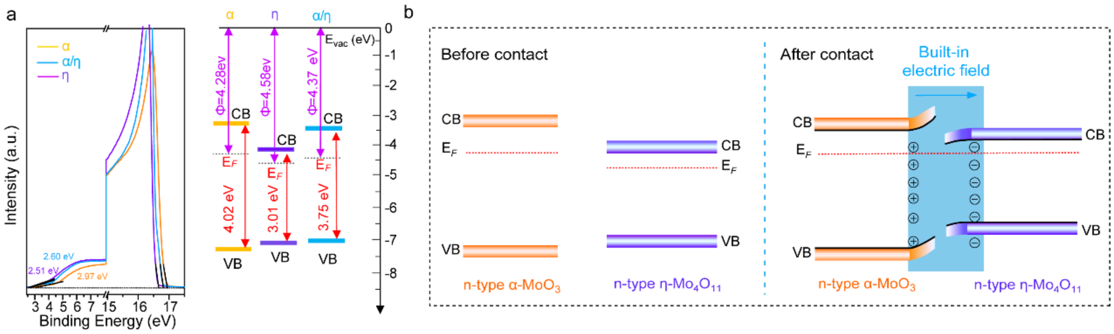

Supplementary Figure 14 Interfacial electronic structure analysis. (a) Valence band spectra and corresponding band structure diagrams determined by ultraviolet photoelectron spectroscopy of η-Mo_4O_{11}, α-MoO_3/η-Mo_4O_{11}, and α-MoO_3 thin films. (b) The schematic illustration of the built-in electric field between n-type α-MoO_3 and n-type η-Mo_4O_{11}.

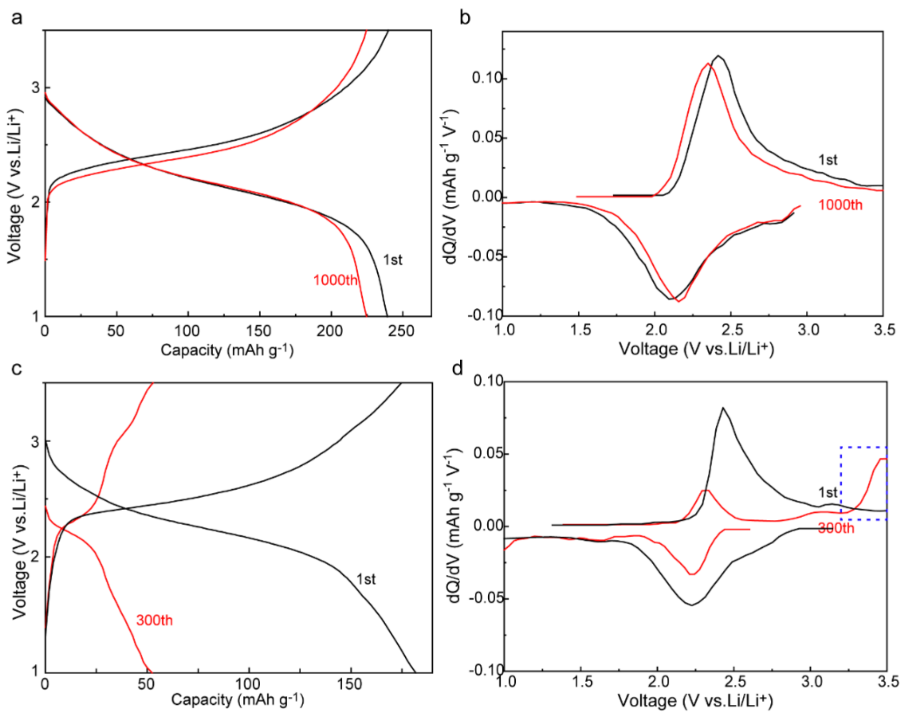

Supplementary Figure 15 The cycling behaviors comparison. Galvanostatic charge/discharge profiles of the (a) α-MoO_3/η-Mo_4O_{11} and (c) α-MoO_3 electrodes collected at different cycle numbers. Calculated dQ/dV profiles of the (b) α-MoO_3/η-Mo_4O_{11} and (d) α-MoO_3 electrodes after different cycle numbers at a specific current of 0.5 A g^{-1}.

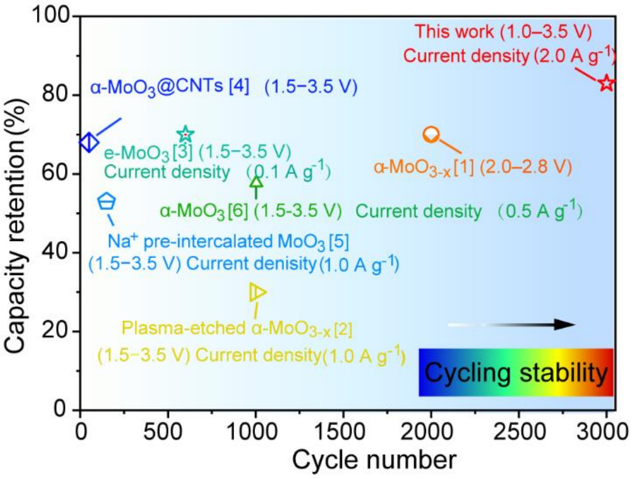

Supplementary Figure 16 Cycle performance comparison between the α-MoO_3/η-Mo_4O_{11} electrode in this work and the reported α-MoO_3-based electrodes in literature.

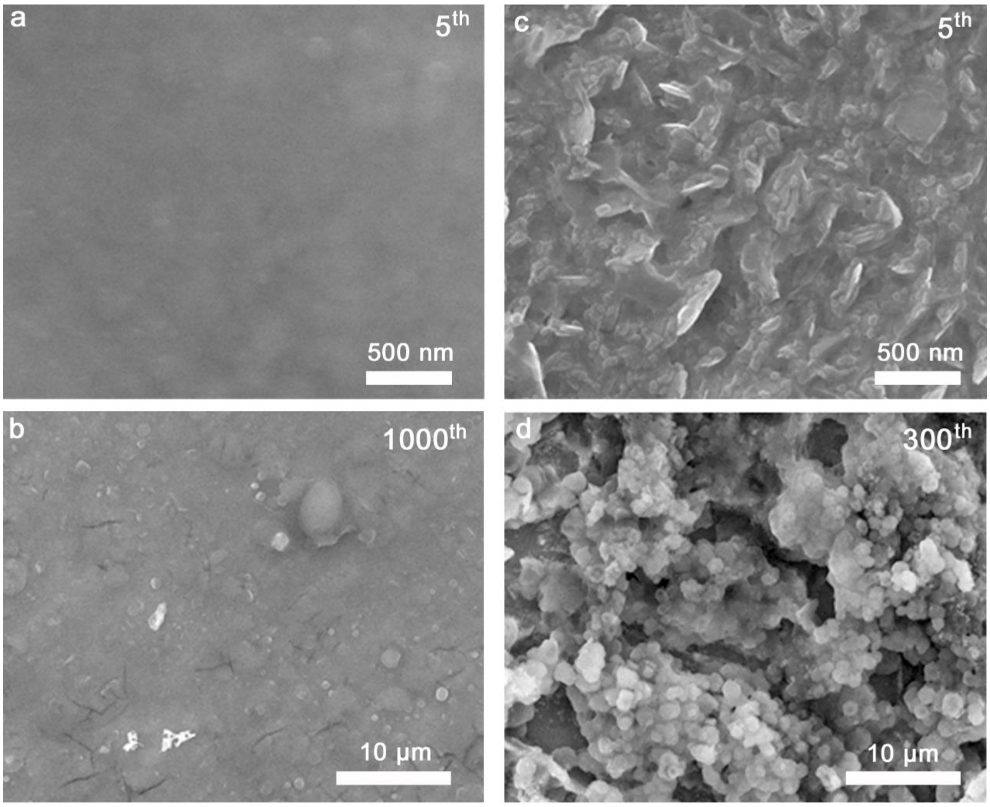

Supplementary Figure 17 Morphology of cycled electrodes. FESEM images of α-MoO_3/η-Mo_4O_{11} thin films collected at the (a) 5th and (b) 1000th cycles. FESEM images of α-MoO_3 thin films collected at the (c) 5th and (d) 300th cycles.

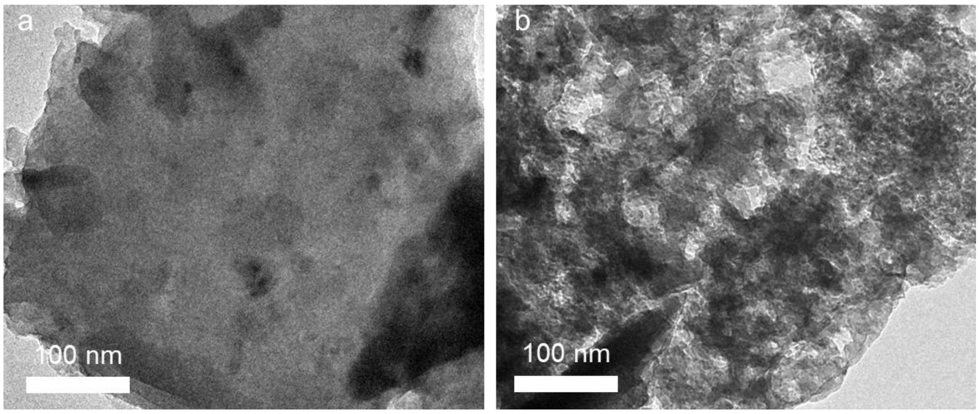

Supplementary Figure 18 Morphology of cycled electrodes. TEM images of (a) α-MoO_3/η-Mo_4O_{11} and (b) α-MoO_3 thin films collected at 5th cycles.

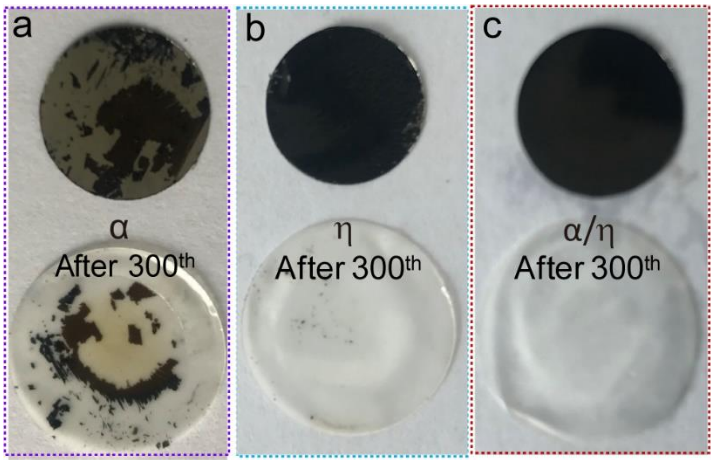

Supplementary Figure 19 The photo images of electrodes after 300 cycles. (a) α-MoO_3 (α), (b) η-Mo_4O_{11} (η), and (c) α-MoO_3/η-Mo_4O_{11} (α/η) thin films.

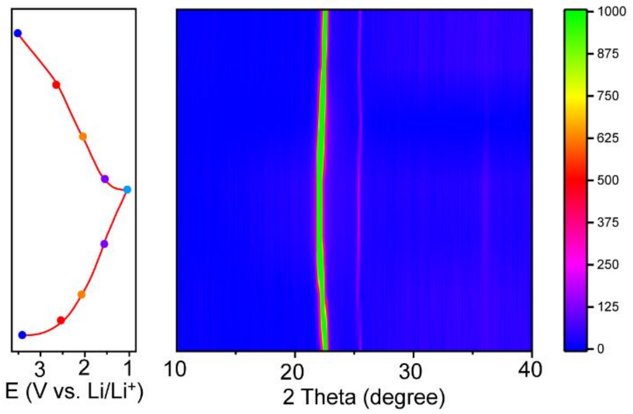

Supplementary Figure 20 Ex situ XRD measurements of the η-Mo_4O_{11} electrode during the first discharge–charge process between 1.0 and 3.5 V (*vs.* Li/Li^+).

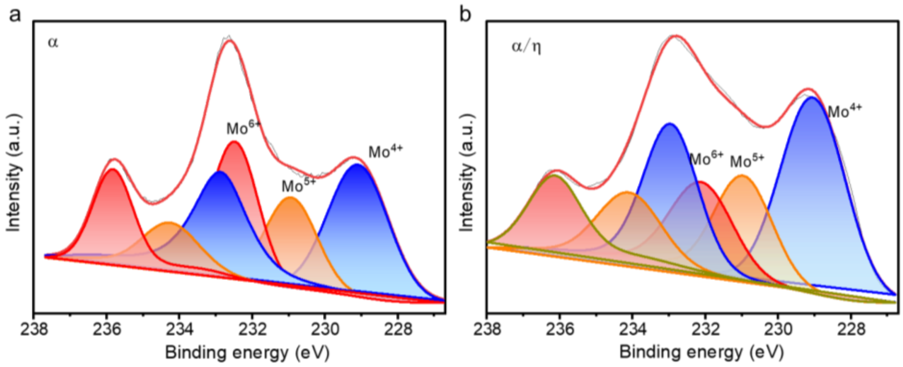

Supplementary Figure 21 Mo *3d* core-level XPS spectra at the lithiated state (1.0 V *vs*. Li/Li^{+}). (a) α-MoO_3 (α) and (b) α-MoO_3/η-Mo_4O_{11} (α/η) electrodes.

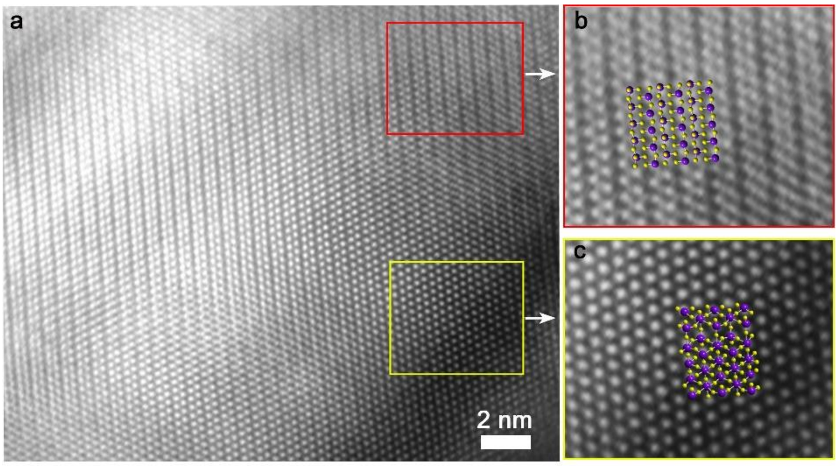

Supplementary Figure 22 Atomic structure of cycled $MoO_3/\eta\text{-}Mo_4O_{11}$ electrode. (a) HAADF-STEM image for the $\alpha\text{-}MoO_3/\eta\text{-}Mo_4O_{11}$ electrode collected after 100 cycles. (b) Atomic arrangement in the red square represents orthorhombic $\alpha\text{-}MoO_3$ along [100] zone axis. (c) Atomic arrangement in the yellow square represents monoclinic $\eta\text{-}Mo_4O_{11}$ along [001] zone axis. The purple spheres represent Mo atoms and the yellow spheres represent oxygen atoms.

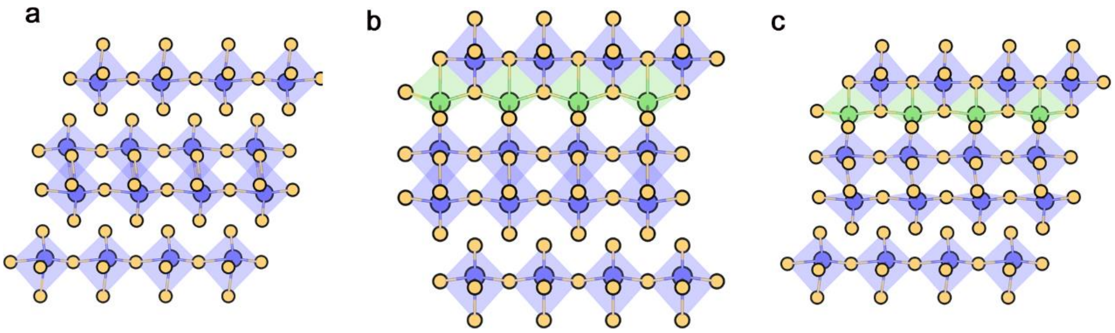

Supplementary Figure 23 Relaxed lattice structures of (a) α-MoO_3, (b) f-Li_xMoO_3, and (c) c-Li_xMoO_3.

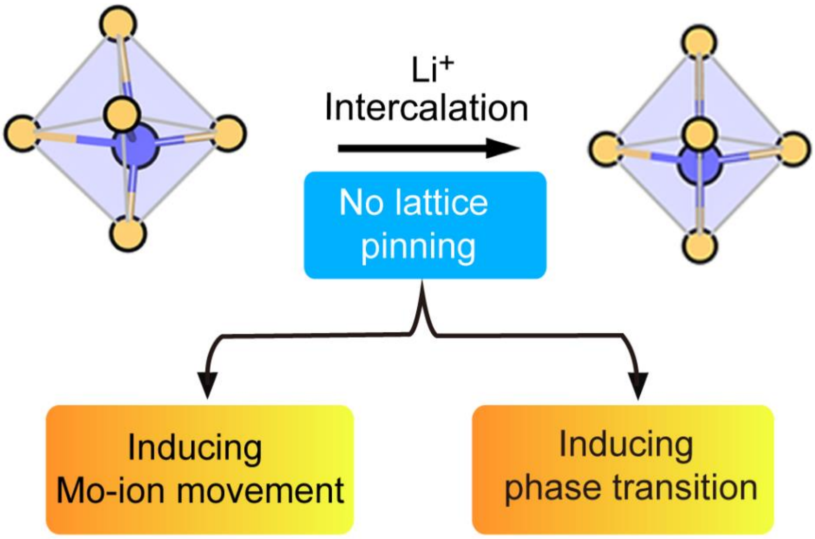

Supplementary Figure 24 α-MoO_3 with no lattice pinning undergoes severe migration of the Mo ions and irreversible phase transition during the Li^+ intercalation process.

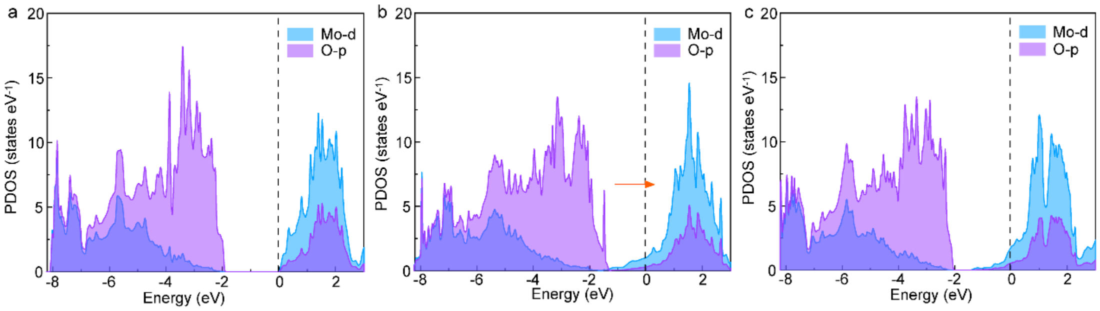

Supplementary Figure 25 Projected density of states (PDOS). (a) α-MoO_3, (b) f-Li_xMoO_3, and (c) c-Li_xMoO_3.

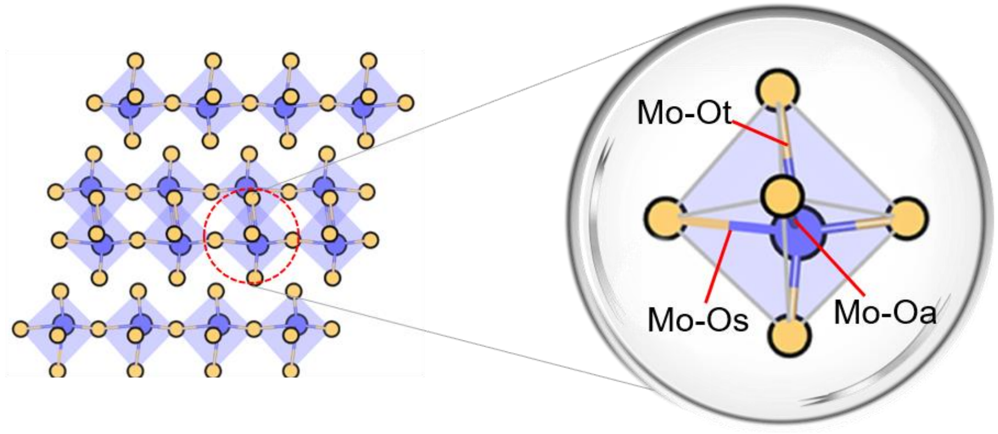

Supplementary Figure 26 Crystal structure of α-MoO_3. The structural framework consists of a bilayer network of edge-sharing MoO_6 octahedra. There are three types of oxygen anion. The 'terminal oxygen' (O_t) results in a Mo-O_t bond that points perpendicular to the vdW gap. The second type, labelled as O_a, forms asymmetric bonds with two Mo ions in the *a* direction, while the third type refers to as 'symmetric oxygen' (O_s).

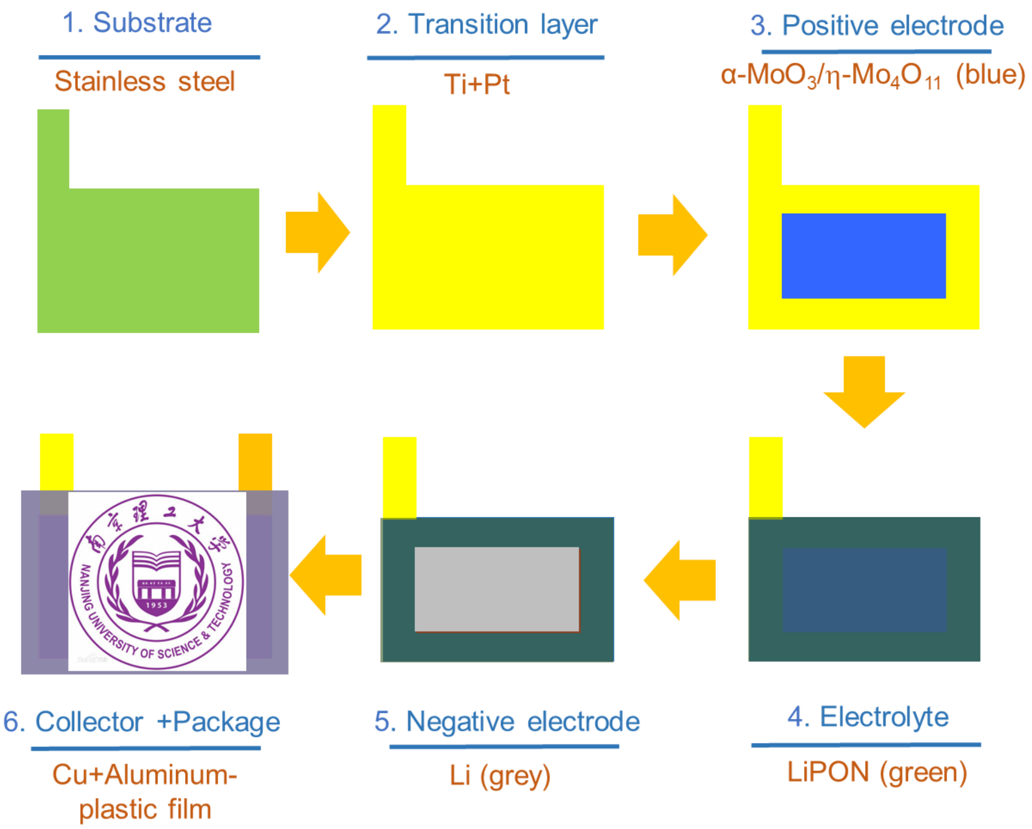

Supplementary Figure 27 The fabricated process of all-solid-state thin-film microbattery.

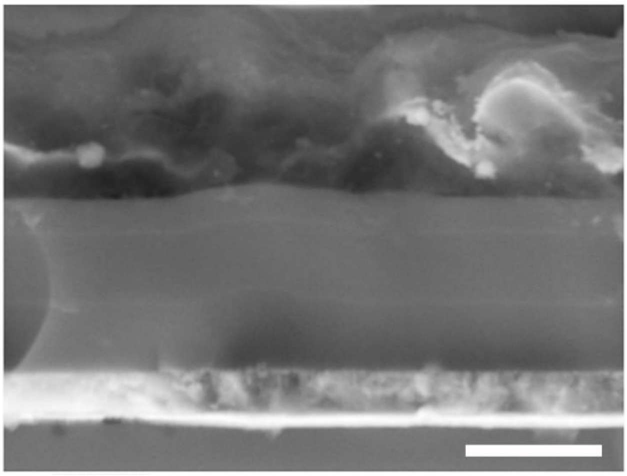

Supplementary Figure 28 Cross-section SEM image of the α-MoO_3/η-Mo_4O_{11}-based all-solid-state lithium microbattery (scale bar, 2 μm).

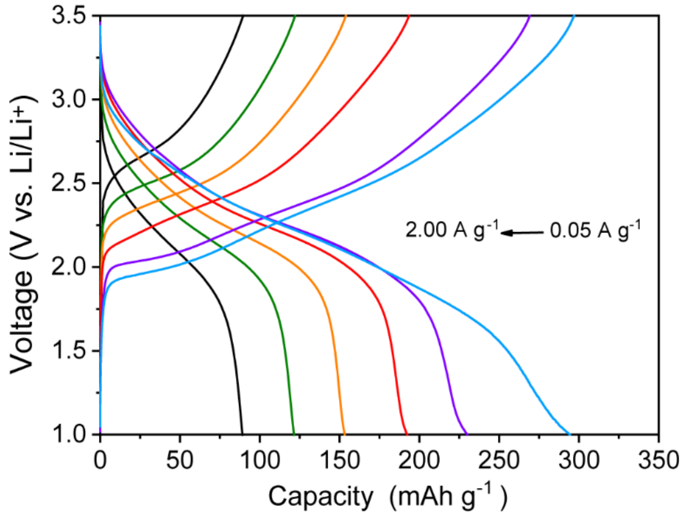

Supplementary Figure 29 Galvanostatic charge/discharge profiles of the α-MoO_3/η-Mo_4O_{11} based all-solid-state lithium microbattery at different specific currents.

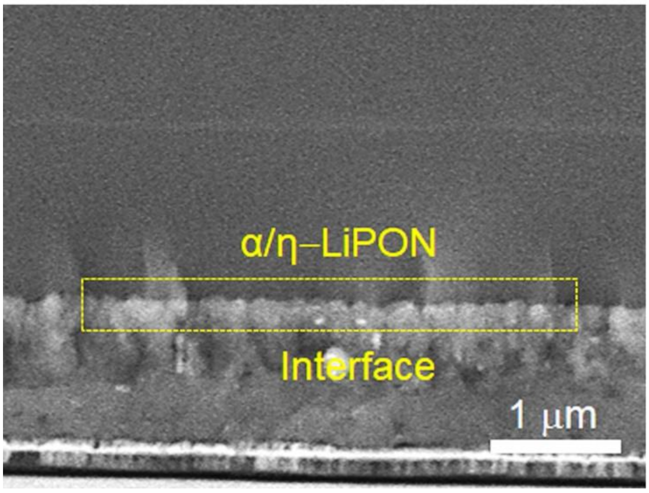

Supplementary Figure 30 Cross-section FESEM image of the α-MoO_3/η-Mo_4O_{11} (α/η)–LiPON interface after cycling at different bending states.

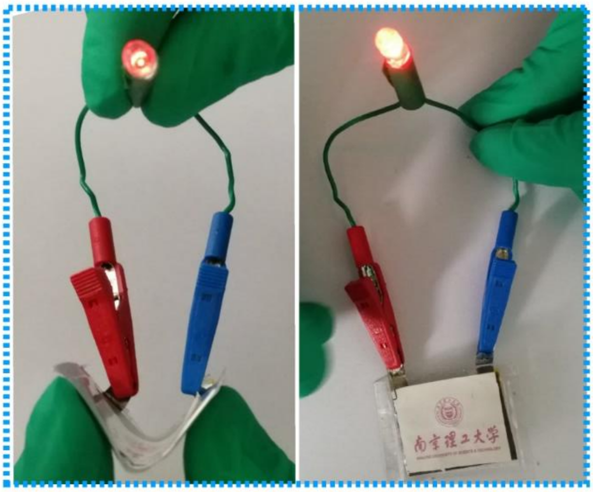

Supplementary Figure 31 Photo images of a sealed all-solid-state lithium microbattery powering a LED under different bending conditions.

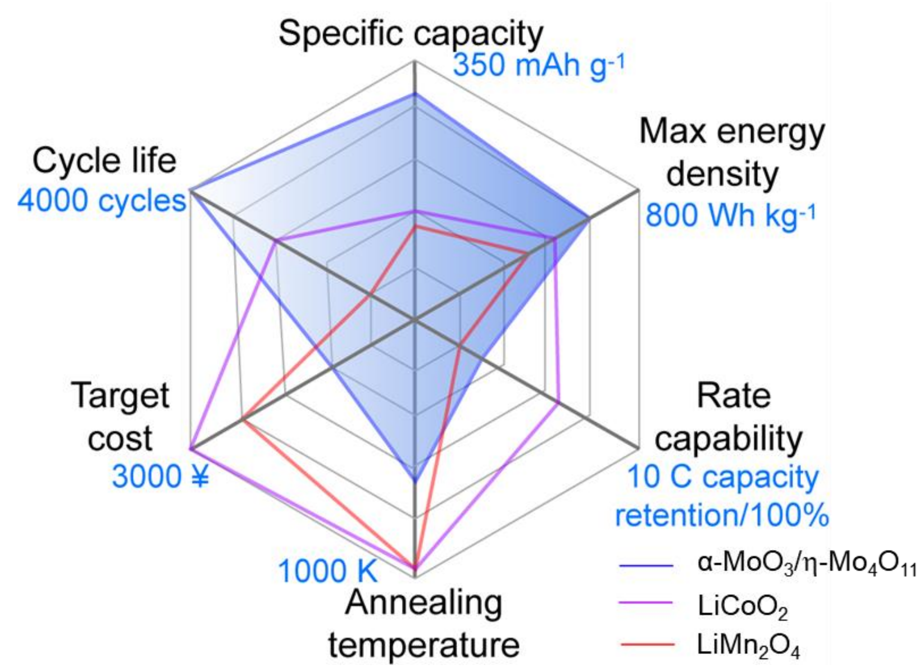

Supplementary Figure 32 Radar plots illustrating crucial metrics to assess α-MoO_3/η-Mo_4O_{11}, $LiMn_2O_4$, and $LiCoO_2$ positive electrodes in all-solid-state batteries.

Supplementary Table 1 ICSD structures used for Rietveld refinement of α-MoO_3, α-MoO_3/η-Mo_4O_{11}, and η-Mo_4O_{11}.

Phase	Space group	ICSD code	Ref.
α-MoO_3	*Pbnm*	36167	7
η-Mo_4O_{11}	$P2_1/a$	15814	8

Supplementary Table 2 The refined crystallographic parameters of the Mo-based electrode materials by the XRD patterns.

Sample	a(Å)	b(Å)	c(Å)	V(Å^3)	R_{wp}(%)	R_{exp}(%)
α-MoO_3	3.9538(4)	13.8944(8)	3.6909(6)	202.7(7)	7.0	8.4
η-Mo_4O_{11}	24.2538(1)	5.6786(5)	6.7210(9)	923.1(1)	9.6	6.9
α-MoO_3/η-Mo_4O_{11} (η)	24.3090(0)	5.6520(1)	6.7867(9)	928.0(8)	8.3	7.0
α-MoO_3/η-Mo_4O_{11} (α)	3.9630(3)	13.9243(7)	3.6942(0)	205.0(4)	8.3	7.0

Supplementary Table 3 Fitting parameters and corresponding errors between the raw and fitted data for EIS spectra of α-MoO_3/η-Mo_4O_{11}, α-MoO_3, and η-Mo_4O_{11} electrodes.

Electrodes	R_s	R_{ct}
α-MoO_3/η-Mo_4O_{11}	1.8 (±1.7%)	129.7 (±2.0%)
α-MoO_3	2.8 (±2.2%)	166.1 (±1.3%)
η-Mo_4O_{11}	2.6 (±0.9%)	247.4 (±1.6%)

Supplementary Table 4 Comparison of the electrochemical performances of α-MoO_3-based positive electrodes in lithium batteries. The double asterisks represent the undefined mass loading.

**Positive electrodes**	**Specific capacity**	**Capacity retention**	**Mass loading**	**Ref.**
R-α-MoO_{3-x}	279 mAh g^{-1}	75% after 1000 cycles	**	1
H_2O-α-MoO_{3-x}	277 mAh g^{-1} at 50 mA g^{-1}	71% after 600 cycles at 100 mA g^{-1}	0.6±0.1 mg cm^{-2}	3
Na^+ pre-intercalated α-MoO_{3-x}	250 mAh g^{-1} at 100 mA g^{-1}	53% after 150 cycles at 500 mA g^{-1}	**	5
Oxygen-deficient α-MoO_{3-x}	225 mAh g^{-1} at 1000 mA g^{-1}	33% after 1000 cycles at 500 mA g^{-1}	**	2
α-MoO_3 nanobelt	290 mAh g^{-1}	50% after 50 cycles	**	4
α-MoO_3 nanobelt@CNTs	175 mAh g^{-1}	65% after 50 cycles	**	4
Single-crystalline α-MoO_3 nanofibers	241 mAh g^{-1}	71% after 30 cycles	**	9
α-MoO_3 thin film	280 mAh g^{-1} at 50 mA g^{-1}	31% after 1000 cycles at 500 mA g^{-1}	0.2 mg cm^{-2}	6

Supplementary Table 5 Comparison of the Mo-O bond among the α-MoO_3, free-Li_xMoO_3, and constrained-Li_xMoO_3.

**α-MoO_3**	**free-Li_xMoO_3**	**constrained-Li_xMoO_3**
Mo-O_t=2.372 Å	Mo-O_t=2.461 Å	Mo-O_t=2.371 Å
Mo-O_a=1.961 Å	Mo-O_a=1.977Å	Mo-O_a=1.957 Å
Mo-O_s=2.185 Å	Mo-O_s=1.935 Å	Mo-O_s=2.186 Å

Supplementary references

1. Kim, H.-S., *et al.* Oxygen vacancies enhance pseudocapacitive charge storage properties of MoO_{3-x}. *Nat. Mater.* **16**, 454–460 (2017).
2. Zhang, G., *et al.* α-MoO_{3-x} by plasma etching with improved capacity and stabilized structure for lithium storage. *Nano Energy* **49**, 555–563 (2018).
3. Yu, M., *et al.* Interlayer gap widened α-phase molybdenum trioxide as high-rate anodes for dual-ion-intercalation energy storage devices. *Nat. Commun.* **11**, 1348 (2020).
4. Lu, W., *et al.* Ultrasonic synthesis of α-MoO_3 Nanobelt@ CNTs composite for lithium battery and its electrochemical performances. *Int. J. Electrochem. Sci.* **13**, 275–286 (2018).
5. Dong, Y., *et al.* Inhibiting effect of Na^+ pre-intercalation in MoO_3 nanobelts with enhanced electrochemical performance. *Nano Energy* **15**, 145–152 (2015).
6. Sun, S., *et al.* Self-standing oxygen-deficient α-MoO_{3-x} nanoflake arrays as 3D cathode for advanced all-solid-state thin film lithium batteries. *Journal of Materiomics* **5**, 229–236 (2019).
7. Nugrahaningtyas, K. D. et al. Synthesis and characterization of CoMo/Mordenite catalyst for hydrotreatment of lignin compound models. *Open Chem.* **17**, 1061–1070 (2019).
8. Tomaszewski, P. E. Structural phase transitions in crystals. I. Database. *Phase Transit.* **38**, 127–220 (1992).
9. Dewangan, K., et al. Synthesis and characterization of single-crystalline α-MoO_3 nanofibers for enhanced Li-ion intercalation applications. *CrystEngComm* **13**, 927–933 (2011).
